# Models of school recess for combatting overweight in the United States

**DOI:** 10.1016/j.pmedr.2022.102081

**Published:** 2022-12-02

**Authors:** David Kahan, Allison Poulos

**Affiliations:** a Spadoni College of Education and Social Sciences, Coastal Carolina University, Conway, SC, USA; b College of Health Solutions, Arizona State University, Phoenix, AZ, USA

## Abstract

**Objectives:**

The aim of the study was to quantify and compare potential energy expenditure associated with school recess in the U.S. based on four scenarios: professional recommendations and state policies for the conduct of recess, previous studies that measured physical activity intensity during recess (i.e., reality), and no daily recess.

**Methods:**

Estimated energy expenditure (kcal) was modeled using secondary data over six years of elementary school for boys and girls using a standard formula: Intensity × duration × frequency × mass .

**Results:**

Boys and girls would expend similar energy under the professional recommendation (boys, 69,146 kcal; girls, 63,993 kcal) and state policy (boys, 69,532 kcal; girls, 64,351 kcal) scenarios. These values are significantly greater than a no recess scenario (boys, 26,974 kcal; girls, 24,821 kcal). The greatest energy expenditure was found for the reality scenario, based on actual studies that measured physical activity intensity (boys, 82,208 kcal; girls, 75,628 kcal).

**Conclusions:**

Professional recommendations and state policies for recess duration may be overly conservative and recommendations for percentage of MVPA may be overly liberal compared to the reality of energy expended during recess. Both potential and real estimates dwarf a scenario of withholding recess (i.e., no recess), which is discouraged in only six state policies. Mandated reporting with “groundtruthing” is needed to determine true recess frequency/duration and state policy compliance.

## Highlights

• Physical activity during elementary school recess has the potential to significantly contribute to daily energy expenditure.
• We modeled recess energy expenditure under four scenarios: professional recommendations, state policies, U.S. research studies that measured dosage and MVPA, and no recess.
• Boys and girls could expend an additional 39,000–55,000 kcal over 6 years by participating in recess compared to no recess.
• Recess duration contributed more to this gap than physical activity intensity.

## 1 Introduction

Robust evidence supports the public health benefits of physical activity (PA) for children, including improved cardiorespiratory, metabolic, and mental health (Janssen and Leblanc, 2010); however, more than 75 % of children in the United States (U.S.) do not meet the recommended 60 min of moderate-to- vigorous-physical activity (MVPA) per day (National Physical Activity Plan Alliance, 2018, Centers for Disease Control and Prevention, 2020). Inadequate PA contributes to insufficient energy expenditure (EE), contributing to weight gain (Remmers et al., 2014) and obesity (Hills et al., 2011) among children. Engagement in regular PA is imperative as obesity rates among U.S. children have risen threefold over the past three decades (Hedley et al., 2004, Ogden et al., 2006), contributing to increased risk of cardiovascular disease (Cote et al., 2013), type 2 diabetes (Bacha and Gidding, 2016), and mental health problems such as anxiety and depression (Halfon et al., 2013).

Schools are widely recognized as critical settings for daily PA because they provide access, structure, and systems to support healthy behaviors and health behavior change (Perry et al., 1992). Schools are the only setting that reach nearly all children (Pate et al., 2006, Sallis et al., 1998, Sallis et al., 2003, Story, 1999), with most children spending almost half of their waking hours at school (about 36 weeks/year) for 12 years (Lounsbery et al., 2013a). In elementary schools, physical education (PE), recess, classroom PA breaks, and other before- and after school programs contribute substantially to MVPA accrual (Lounsbery et al., 2013b, Payne and Morrow, 2009, Sallis et al., 2012, Story et al., 2009); however, recess may be the most significant source of PA at school as movement during recess provides up to 44 % of all school-based PA (Erwin et al., 2012) and counters sedentary time (Guinhouya et al., 2009, Ridgers et al., 2005). Despite the potential, the actual and potential public health impact of PA during recess on levels of children’s overweight and obesity is not clear.

Numerous health organizations, including Centers for Disease Control and Prevention (CDC, 2011) and Society of Health and Physical Educators (SHAPE America) (CDC and SHAPE America, 2017), recommend 20 min or more of daily recess in schools. Across the U.S., most (83 %) elementary schools provide one daily recess period that meets or exceeds the recommended 20-min duration (Clevenger et al., 2022, US Department of Health and Human Services USDHHS/CDC, 2015); however, movement during recess varies and is dependent on factors such as student sex and quality of recess (Reilly et al., 2016). Elementary school boys and girls accrue an average of 1268 and 914 steps, respectively, during recess (Guinhouya et al., 2009). Globally, boys tend to accrue more MVPA minutes than girls during recess; however, quantification of this difference across studies has not been reported (Reilly et al., 2016). Offering quality recess by incorporating strategies such as adding equipment or enhancing the playground environment impacts movement (Parrish et al., 2013, Reilly et al., 2016, Ridgers et al., 2012a) and can add an additional five and six minutes, respectively, to children’s MVPA time during recess (Bassett et al., 2013).

The development of school-based policy is a public health strategy that impacts both the provision and quality of recess (Whitehouse and Schafer, 2017). State policy predicts the likelihood of having a district policy that supports PA, acting as a policy ‘floor’ to set the stage for PA support (Chriqui et al., 2020). For example, schools in states with recess mandates are 1.8x more likely to provide the recommended 20 min of daily recess (Slater et al., 2012). Withholding recess for punishment or academic reasons remains a widespread barrier in the U.S. (Murray and Ramstetter, 2013); however, schools are less likely to keep students from recess when district policies preventing the withholding of recess exists (Turner et al., 2013). State-level policies in support of recess have increased over the last decade, but the strength of policies varies. To support effective policy- and decision-making to promote quality recess, the purpose of this paper is to assess the potential impact that PA during recess has, and can have, on energy balance.

PA can be classified by intensity, duration, frequency, and type – all of which can be used to determine energy expenditure. In the scientific community energy expenditure is typically expressed as metabolic equivalents of task (MET), or the energy costs associated with physical activities. MET values provide common scientific representations of PA volume by multiplying the energy expenditures of activities (MET values) by the duration. Values of 5.7–5.9 MET_y_ are afforded to PA in the context of freeplay, which is commonly performed at recess (Butte et al., 2018). However, in the medical and lay communities, METs are either not used or readily comprehensible. Instead, calories (i.e., kcal) is the term most are familiar with or comfortable using. To determine the public health impact of recess, the degree to which MVPA increases energy expenditure (and/or reduces caloric intake) is needed. Moreover, identifying energy expenditure in kcal can broaden the conversation about PA and its relationship to overweight and obesity to include persons who can ultimately drive efforts that ensure provision of quality (i.e., of sufficient duration and intensity) recess. The aim of the study was to assess both the potential and actual energy expenditure for recess across six years under four scenarios:

1. Current professional recommendations (i.e., potential),

2. Existing state policies (i.e., potential),

3. Actual studies reporting recess intensity and duration (i.e., reality), and.

4. No daily recess.

## 2 Methods

**Fig. 1:**
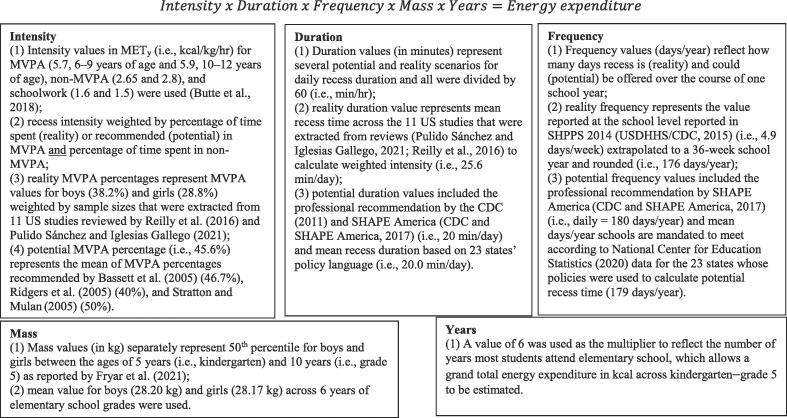
Exposition and Formula of Component Variables and their Sources for Computing Recess Energy Expenditure in Kcal: United States, 2022.

We utilized the simulation methods that Kahan and McKenzie (2017) employed to calculate actual and potential energy expenditure in PE. Secondary data were used to estimate energy expenditure (kcal) among boys and girls averaged over six years of elementary school using a standard formula: Intensity × duration × frequency × mass (Fig. 1). Data were obtained from various sources (explained below) to align with each of our four scenarios (professional recommendations, state policy, actual studies, and no daily recess) in June 2022. These data were available from publicly available sources, and thus exempt from ethical compliance (SDSU, “Not Subject to IRB Review Determination,” October 17, 2022).

### 2.1 Energy expenditure sources and calculations

Overall, we followed the guidance of Butte et al. (2018) who stated: “An estimate of the energy cost of a physical activity can be computed based on the MET_y_ value from the Youth Compendium, a measured or computed BMR, and duration of the specific activity as follows: energy cost (kcal) = MET_y_ × BMR (kcal•min^−1^) × duration (min)” (p. 53). As such, we accounted for children’s sex-specific BMR by using the Schofield equations (Schofield et al., 1985).

#### 2.1.1 Intensity

We obtained MET PA values and percentage of time spent in MVPA during recess to calculate intensity. For potential and real scenarios, we utilized 5.7 and 5.9 MET_y_ to represent MVPA during recess for children ages 6–9 and 10–12 years, respectively (Butte et al., 2018). These two values, extracted from the Youth Compendium (Butte et al., 2018), are ascribed MET_y_ codes 101602 and 101603, respectively, and are classified under activity category “active play” and specific category “free play (basketball, rope, hoop, climb, ladder, frisbee).” Because no available published research identified MET values for non-MVPA during recess, we assigned a value of 2.65 and 2.8 METy for children ages 6–9 and 10–12 years, respectively, which reflects the average intensity of “walk self-paced casual” (MET_y_ code 80320x) and “standing” (MET_y_ code 70200x) (Butte et al., 2018). To estimate energy expenditure in a scenario where students are deprived of recess, we used the MET_y_ value associated with schoolwork (MET_y_ code 55400x; 1.6 MET and 1.5 MET for children ages 6–9 and 10–12 years, respectively) (Butte et al., 2018).

To calculate MVPA percentage for potential scenarios, we averaged the percent of recess time to be spent in MVPA recommended by three studies’ (45.6 %) (Bassett et al., 2013, Ridgers et al., 2005, Stratton and Mullan, 2005). For real energy expenditure estimates, we consulted various systematic reviews of objectively measured PA during recess (Pulido Sánchez and Iglesias Gallego, 2021, Reilly et al., 2016, Ridgers et al., 2005, Ridgers et al., 2012a); however, none reported a single, weighted percentage value of recess MVPA, and we were unsuccessful in acquiring this information from the authors. Therefore, we identified studies of elementary school children conducted in the U.S. that appeared in the latest two reviews (i.e., Pulido Sánchez and Iglesias Gallego, 2021, Reilly et al., 2016); extracted the reported MVPA percentages for boys and girls or calculated the percentages when minutes of MVPA and recess duration were reported instead; and weighted each study’s MVPA percentages by the reported number of male and female participants. The resulting MVPA percentages for boys (38.2 %) and girls (28.8 %) represented 11 studies of 1231 boys and 1243 girls. We extracted the following additional contextual information – when it was reported – from the 11 studies: (1) number of schools sampled per study (range, 1–12); (2) objective measures of physical activity (uniaxial accelerometry (*n* = 8), systematic observation (*n* = 4), triaxial accelerometry (*n* = 2), heartrate telemetry, pedometry (*n* = 1 each)); (3) racial composition of participants (range, 13 %–95 % White); (4) age of participants (range of means, 8.9–11.0 years); (5) BMI of participants (range of means, 18.5–20.4; 52 %–78 % normal weight); and (6) percent economically disadvantaged (range, 31 %–85 %).

#### 2.1.2 Duration

For the potential professional recommendation scenario, we utilized guidance from the CDC (CDC, 2011) and SHAPE America (CDC and SHAPE America, 2017). For the potential policy scenario, we calculated the mean state policy recess duration using data from the National Association of State Boards of Education (National Association of State Boards of Education, n.d.), which outlines each state’s recess policy based on specificity and strength. We differentiated state policies exclusive to recess and those that comingle recess with other forms of PA. For the latter, we partitioned recess (or unstructured play) minutes from other PA forms. For example, South Carolina requires 90 min/week of PA that can include PE or recess; thus, we halved 90 (i.e., 45) because policy allows for one or the other, then divided by 5 days to arrive at nine min/day of recess (Table 1). For the real energy expenditure scenario, we calculated mean minutes (25.6 min) reported in the 11 U.S. studies used for identifying recess intensity. We considered using recess duration reported by schools in the School Health Policy and Practice Study (SHPPS) 2014 (26.9 min) (USDHHS/CDC, 2015), but elected to use the more recent group of published studies as they reported objective PA data.

**Table 1:** State Policies and School Characteristics for Calculating State Policy-Based, Potential Recess Energy Expenditure for Elementary School Students (*n* = 23): United States, 2022.

State	Policy language (specificity)	Strength	Min/day	School days/year
States with policy language focused solely on recess				
GA	At least 20 min/day recess	Recommends	20	180
HI	At least 20 min/day recess	Recommends	20	180
IL	30 min/day recess	Recommends	30	185
IN	At least one 20-min period/day active recess	Requires	20	180
KS	15 min/day recess	Recommends	15	186
MO	20 min/day recess	Requires	20	—
NV	At least 20 min/day recess	Recommends	20	180
NJ	At least 20 min/day recess	Requires	20	180
NY	At least 20 min/day recess	Strongly recommends	20	180
NC	At least 30 min/day recess	Requires	30	185
OK	At least 20 min/day recess	Strongly recommends	20	180
RI	20 consecutive min/day recess	Requires	20	180
UT	20 min/day recess	Considers best practice	20	180
WV	30 min/day recess	Requires	30	180
States with policy language mixing *recess* with other physical activity (PA)				
AK	54 min/day of PA…can include PE, *recess*, and/or schoolwide recreation	Must	18	180
AR	40 min/day *recess* and unstructured play	Requires	40	—
CO	600 min/month of PA…can include PE, fitness breaks, *recess*, and/or field trips	Must	7.5	160
FL	At least 100 min/week unstructured play/*recess*	Requires	20	180
IA	At least 30 min/day of PA…can include *recess*, gym class, brain breaks, etc.	Requires	10	180
LA	No less than 30 min/day x 5 days/week of moderate PA and 20 min/day x 3 days/week of vigorous PA…can include PE, *recess*, etc.	Requires	21	177
SC	At least 90 min/week of PA…can include PE or *recess*	Must	9	180
TX	At least 30 min/day of MVPA… as part of PE or daily *recess*	Requires	15	—
VT	At least 30 min of PA…can include *recess* or a movement-based curriculum	Strongly recommends	15	175
Mean (SD)			20.0 (7.1)	179.4 (5.1)

*Note*. — = no state level requirement for minimum days of instruction.

#### 2.1.3 Frequency

For the potential professional recommendation scenario, we utilized the value of 180 days/year recommended by SHAPE America. For the potential policy scenario, we used 179 days/year which represents the rounded value of the mean number of days in a school year for the 23 states with recess time policies (Table 1) reported by the National Center for Education Statistics (National Center for Education Statistics, 2020). For real energy expenditure estimates, we used the SHPPS 2014 value of 4.9 days/week of recess, which extrapolates to 176 days/year (USDHHS/CDC, 2015).

#### 2.1.4 Mass

We separately calculated mean mass of boys and girls ages 5–10 years based on the most recent anthropometric data on US children (Fryar et al., 2021). The mean mass for boys (28.2 kg) was only 0.12 percent greater than girls’ mass.

## 3 Results

**Fig. 2:**
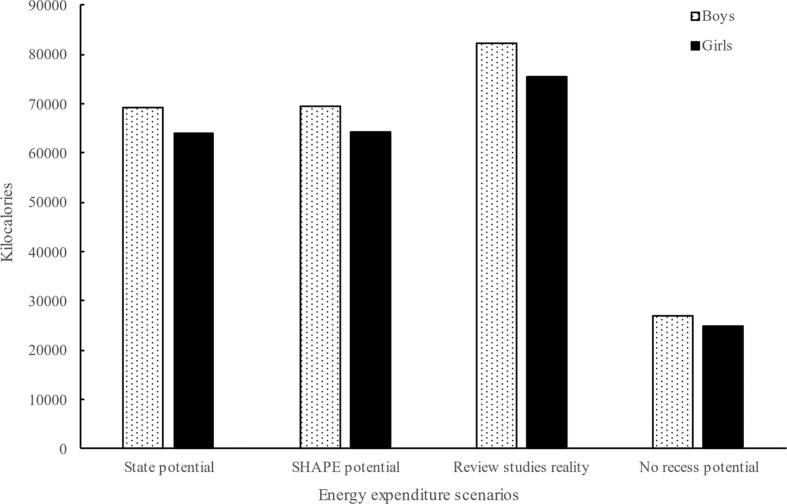
Graphical Representation of Estimated Energy Expenditure during Recess under Various Scenarios: United States, 2022.

Energy expenditure estimates under potential recess scenarios were similar between professional and policy conditions (Fig. 2). Specifically, boys and girls would expend 69,532 kcal and 64,531 kcal based on professional recommendation (i.e., 20 min/day, 180 days/year) and 69,146 kcal and 63,993 kcal based on the average of state policy conditions (i.e., 20.0 min/day, 179 days/year), respectively (Fig. 2). Energy expenditure estimates under the reality scenario were higher than under both potential scenarios: boys (82,208 kcal) and girls (75,629 Kcal) (Fig. 2). Sex differences for energy expenditure resulted from differences in BMR calculations and were more pronounced under the reality scenario due to the different percentages of MVPA accrued by boys (38.2 %) vs girls (28.8 %). By comparison, if recess was withheld and the identical time spent doing seated schoolwork, boys and girls would expend 26,974 kcal and 24,821 kcal, respectively (Fig. 2). The values estimated for the no recess scenario are approximately 61 % less than professional recommendation and state policy scenarios, and approximately 67 % less than the reality scenario.

## 4 Discussion

Our results demonstrate that recess under potential (professional recommendations and/or state policies) and reality scenarios can significantly impact energy expenditure beyond a scenario where recess is not offered, or an equivalent interval of schoolwork. Specifically, over six years of elementary school, boys and girls could expend an additional 42,172–55,234 kcal and 39,172–50,808 kcal, respectively, in recess compared to having none. Given that our estimates of energy expenditure using data from real research studies were higher than estimates using both professional recommendations and state policies, there is a need to consider whether current guidance for recess duration may be overly conservative and percentage of time in MVPA may be overly liberal compared to the reality of energy expended during recess.

Recess is a prominent source of PA accessible by most children, but the provision of recess is not equitable across the U.S. Despite recommendations from numerous national organizations that recess not be withheld (e.g., American Academy of Pediatrics (Council on School Health, 2013); SHAPE America (SHAPE America, 2021)), the practice of keeping students from recess for academic reasons or punishment, or using PA during recess as behavior management, still occurs in schools (Murray and Ramstetter, 2013, Turner et al., 2013). State policy can facilitate opportunities for adequate PA at school (Sallis et al., 1998, Whitehouse and Schafer, 2017). School districts with policies that prevent the withholding of recess are twice less likely to keep students from recess (Turner et al., 2013). Although there is a growing momentum across the U.S. to enact laws in favor of recess, the strength of policies varies widely. Currently, 23 states have either a codified or non-codified law that supports recess. Of those, 11 states require recess and only two (NJ and RI) require recess and prohibit its withholding (National Association of State Boards of Education, n.d.). As state level policies continue to grow in support of recess, there should be an emphasis on strong language and compliance because the strength of a state's policy impacts school level practices (Slater et al., 2012). Indeed, there was wide variation in our examination of state policy language. Of the 23 included policies, 14 states used non-specific language (i.e., at least, maximum, minimum) concerning minutes of recess or PA and nine states co-mingled recess with other forms of PA such that recess duration could only be inferred (Table 1). Rhode Island was most specific by identifying a finite recess duration and frequency and qualifying that the time (20 min) be continuous (Table 1). Colorado, in comparison, was least specific by identifying physical activity time allocation by the month (not the day) and comingling recess among four physical activities including field trips (Table 1). (Colorado, to its credit, has a codified policy against withholding recess.) There was similar variation in state policy language strength with 14 states using the word “require” or “must” (Table 1).

Educational policy decisions fall under state auspice in the U.S., resulting in non-uniform policy toward provision of recess. Moreover, with accountability and enforcement of mandates lacking, the true impact of recess on energy expenditure cannot be known with certainty. Where mandates exist, although unpalatable, one avenue for redressing insufficiencies is through litigation. Indeed, in California, successful litigation against 37 school districts for inadequate provision (i.e., providing less than state-mandated 200 min/10 days) of PE in elementary schools, resulted in increased PE minutes and increased achievement of cardiorespiratory fitness standards (Thompson et al., 2018, Thompson et al., 2019). Yet this route is costly, time-consuming, and reactionary. Continued activism and advocacy efforts that recruit and persuade policymakers and gatekeepers to champion recess, while also time-consuming, offer a proactive path toward institutionalizing recess time.

Our potential energy expenditure estimates, unfortunately, cannot be compared to analogs calculated for PE. In their calculations for PE, Kahan and McKenzie (2017) utilized an MVPA value of 4.5 METs; did not utilize youth compendium MET_y_ values nor factor in age-related differences in MET_y_ values, both of which were published after their study; and did not factor BMR into calculations. Although the purposes of PE and recess vastly differ; minimally, recess could be considered an adjuvant to PE in terms of energy expenditure. Moreover, recess may be comparatively more feasible to offer than PE, which requires paying salaries to trained teachers, competing against academic subjects for viability, incurring higher costs for specialized equipment, and overcoming managerial issues associated with motivating individuals to participate in a structured setting.

Recess is a crucial component for healthy childhood development that should be accessible to all (Council on School Health, 2013). It is concerning that in elementary schools, male-, Black-, and disabled students lose 2.1–6.0 times more school days/year due to suspension than female-, White-, and non-disabled students, respectively (Losen and Martinez, 2020). Compounding this differential treatment is that males and Blacks ages 6–11 years have higher obesity prevalence by 3.5 % and 5.8 %, respectively, than females and Whites (Ogden et al., 2018); and compared to non-disabled youth ages 10–17 years, disabled youth have higher obesity prevalence by 6.2 % (hearing/vision condition) to 11.2 % (autism) (Chen et al., 2010). Thus, those at greatest risk of obesity are more likely to lose out on receiving maximal recess dosage across their elementary school years.

### 4.1 Strengths and limitations

Our study is the first, to our knowledge, to quantify the actual and potential energy expenditure of recess in kilocalories but note that our estimations were calculated using data from different studies. In our study, real estimates of energy expenditure were based on MVPA percentages reported in U.S. studies and differentiated by sex differences associated with MVPA accumulation. We note that the inclusion of international studies for deriving recess MVPA percentage and duration would have resulted in even higher energy expenditure estimates. This balloon effect would have been due to a 10-minute increase in recess duration across studies even though MVPA percentages would be 9.0 % and 7.5 % less among boys and girls, respectively, than the U.S. studies alone. Our estimation across six years assumed that energy expenditure remains consistent among children throughout elementary school; however, MVPA during recess generally decreases with age (Grao-Cruces et al., 2019, Ridgers et al., 2012b). Additional studies using longitudinal designs would be beneficial to determine potential energy expenditures specific to elementary children over time.

## 5 Conclusions and directions for future work

We found that recess is an important source of energy expenditure for elementary children in the U.S. Specifically, under potential (professional recommendations and/or state policies) and reality scenarios, children included in our study expended vastly greater energy beyond a scenario where recess is not offered, or an equivalent interval of schoolwork.

Given that the accuracy of our estimates is limited by the quality of available data for input, it is important to undertake regular surveillance – using objective measures – of recess MVPA, duration, and frequency. As well, studies should transparently report recess duration and MVPA data overall and stratified by sex, and separately by condition (i.e., baseline vs treatment). Meanwhile, our study and commentary suggest that between recess duration and intensity, it is duration that has a greater effect on energy expenditure. In turn, a future focus on duration may be more tenable than on intensity, which can be inferred from professional organization and state policy language that exclusively cites the former and omits mention of the latter. Additional studies on the energy expenditure contributions of other components of a comprehensive school physical activity program (CDC and SHAPE America, 2013), such as active transportation to/from school and classroom activity breaks, are warranted.

The idea that children may compensate MVPA during the school day with reduced MVPA out of school has not been supported (Long et al., 2013). Thus, it is incumbent upon schools to maximize PA opportunities for their students. Interventions that increased the overall duration of daily recess generally resulted in increased MVPA (Siedentop, 2009). Increasing the frequency of daily recess periods also resulted in increased MVPA (Kobel et al., 2015). In sum, therefore, increasing recess dosage (i.e., duration × frequency) may be a low-cost, accessible, and sustainable opportunity to increase energy expenditure at school and positively affect children’s health at the population level.

## CRediT authorship contribution statement

**David Kahan:** Conceptualization, Data curation, Formal analysis, Methodology, Writing – original draft, Writing – review & editing. **Allison Poulos:** Data curation, Methodology, Writing – original draft, Writing – review & editing.

## Declaration of Competing Interest

The authors declare that they have no known competing financial interests or personal relationships that could have appeared to influence the work reported in this paper.

## Data Availability

Data will be made available on request.
